# Validity of Using Tuberculin Skin Test Erythema Measurement for Contact Investigation during a Tuberculosis Outbreak in Schoolchildren Previously Vaccinated with BCG

**DOI:** 10.2188/jea.15.56

**Published:** 2005-05-10

**Authors:** Aira Toivgoogiin, Makoto Toyota, Nobufumi Yasuda, Hiroshi Ohara

**Affiliations:** 1 Department of Public Health, Kochi Medical School.; 2 Kochi City Health Center (Present affiliation: Kochi Prefecture Hata Health Center).

**Keywords:** BCG, erythema, induration, tuberculin test, tuberculosis

## Abstract

BACKGROUND: Few studies have examined the validity of administering tuberculosis control measures based on tuberculin skin test (TST) erythema measurement. The present study aimed to clarify the relationship between the erythema and the induration seen following TST and to evaluate the validity of diagnosing tuberculosis infection based on the erythema following TST in school-aged contacts who had been vaccinated with bacillus Calmette-Guérin (BCG) in infancy.

METHODS: A 56-month longitudinal study from January 1999 through September 2003 followed 566 junior high school students in Kochi City who were contacts of an infectious tuberculosis case. To evaluate the diagnostic accuracy of the erythema and induration following TST of the contacts, false-positive and false-negative TST results were noted.

RESULTS: The natural logarithm of the erythematous response size was linearly related to the induration size. When the size of the erythematous response was used to determine the presence of tuberculosis infection, the proportion of infected children increased with increasing exposure to the index case. When the TST results in the contact investigation were interpreted together with the change in the size of the erythematous response from that observed at the regular school-entry checkup, false positive test results were avoidable among the students who had a large erythematous response after the contact investigation TST, but whose response was only slightly larger than their erythematous response following the school-entry TST. Among the students whose TST results were negative, 1.9% developed tuberculosis.

CONCLUSION: Both erythema and induration measurement were equally effective for identifying tuberculosis infection in schoolchildren vaccinated with BCG.

The incidence of tuberculosis in Japan still continues to be higher than that in other developed countries. In 2002 in Japan, the annual notification rate of new tuberculosis (25.8 per 100,000 population) was about 5 times that in Sweden (4.5 per 100,000 population) and the United States (5.6 per 100,000 population).^1^ In Japan there is a large difference in the incidence between the 0-19 year age group and the 20 and older age group; the annual notification rate in 2002 was lower than 5 per 100,000 persons in the younger age group and higher than 15 per 100,000 persons in the older age group.^1^^,^^2^ Therefore, effective measures that prevent the transmission of tuberculosis infection to susceptible children must be promoted.

In the tuberculosis prevention and control program for infants and children in Japan, all infants are inoculated with the bacillus Calmette-Guérin (BCG). The program is based on the effectiveness of BCG vaccination for protecting against disseminated tuberculosis and meningitis in children.^3^ Under the previous version of the Tuberculosis Prevention Law, the BCG revaccination program with repeated tuberculin tests was administered to schoolchildren. The revaccination program had been controversial because there is: (1) a very low risk for tuberculosis infection in children; (2) a lack of evidence supporting the effectiveness of BCG revaccination as a countermeasure to prevent active tuberculosis among infected children;^2^ and (3) the fact that tuberculin skin tests boost cell-mediated immunity which wanes with the passage of time following the initial BCG vaccination.^4^^,^^5^ Hence, the BCG revaccination program for schoolchildren was abolished in 2003. After the abolition of the BCG revaccination program, the tuberculosis control program gave priority to active case finding by targeting contacts of an infectious tuberculosis patient. When an outbreak of tuberculosis infection is suspected to have occurred among schoolchildren, a local public health center conducts a contact investigation, and children with latent tuberculosis infection are referred for chemoprophylaxis to prevent active tuberculosis.^6^^,^^7^

The tuberculin skin test (TST) is widely used in contact investigations to determine the presence or absence of tuberculosis infection. The TST is based on the fact that infection with *Mycobacterium tuberculosis* produces a delayed-type hypersensitivity reaction to tuberculin. The reactions observed at the skin-test site are erythema and induration.^8^^-^^10^ Induration measurement is the international standard for interpreting TST results.^10^ On the other hand, erythema measurement plays an important role both clinically and epidemiologically in Japan because the Tuberculosis Prevention Law requires the notification of the tuberculosis case to include erythema measurement, and induration measurement has great variability when unskilled examiners perform the measurement.^8^^,^^10^ However, a few epidemiologic studies have examined the validity of administering a tuberculosis prevention and control program based solely on erythema measurement.^9^ Unfortunately, lack of evidence for relying solely on erythema measurement distorts the generalizability of Japanese data to the antituberculosis programs of other countries.

The aim of the present study was to examine the validity of interpreting TST results based on erythema measurement. The validity was evaluated from two points of view: the relationship between the degrees of erythema and induration, and the diagnostic accuracy of erythema measurement done during a contact investigation for tuberculosis infection. The data set was obtained from a 56-month longitudinal study done in a junior high school which followed contacts of a student with infectious tuberculosis.

## METHODS

### Study subjects

The index case was a 15-year-old, female, third-grade student in a Kochi City junior high school. Details of her clinical characteristics were described in our previous reports.^11^^,^^12^ She developed a persistent cough at the beginning of December 1998, and was identified as having sputum smear-positive, cavitary pulmonary tuberculosis on January 28, 1999. The municipal public health center conducted a contact investigation for 616 contacts (three family members, 566 students, and 47 teachers) in March 1999. The contacts were examined with periodic screening (semiannual chest X-ray during the first two years and a chest X-ray in the third year) until March 2002. Tuberculosis cases that occurred from April 2002 through September 2003 were identified based on the records of the registry of tuberculosis disease kept by the public health center. All of the contacts and their parents were told about the municipal public health center’s role as the local authority in administering the tuberculosis prevention and control program and gave their consent to participate in the contact investigation and to be followed with regard to the occurrence of tuberculosis disease.

During the 56-month follow-up period from January 1999 through September 2003, 34 secondary cases (three family members, 24 students, six teachers, and one contact that was not part of the school population) were identified; of these, 13 cases (two family members, nine students, one teacher, and one contact that was not part of the school population) were bacteriologically positive. According to restriction-fragment-length-polymorphism analysis, the *M. tuberculosis* isolates from 10 culture-positive patients (one family member, seven students, one teacher, and one contact who was not part of the school population) had the same pattern as that of the index case.

Three family members, 47 teachers, and one contact who was not part of the school population were excluded in the present analysis because most of them were aged 30 years or older and did not undergo a TST during the contact investigation. The remainder (566 students) were stratified into 4 groups of decreasing degrees of exposure to the index case, based on proximity and shared school activities: group 1, 30 homeroom classmates; group 2, 72 students (five first-grade students, seven second-grade students, and 60 third-grade students) who shared special classes or extracurricular activities with the case; group 3, the remaining 104 third-grade students; and group 4, the remaining 360 students of the other school grades (183 first-grade students and 177 second-grade students). Students of groups 1-3 were examined with chest X-ray done by the public health center immediately after the notification of the index case. Two students (one in group 1, the other in group 2) were found to have chest X-ray findings consistent with tuberculosis. In March 1999 (two months after the notification of the index case), 543 students (29 in group 1, 71 in group 2, 99 in group 3, and 360 in group 4) had a TST done at the public health center, and 21 students had TSTs at medical facilities. Students who had positive TST results underwent chest X-ray examination at the public health center. Of the 564 students, nine students had findings consistent with pulmonary tuberculosis; 144 students were considered to have latent tuberculosis infection and were referred for chemoprophylaxis; and 406 students had negative results on the TST and were followed with periodic screening. Of the 144 students referred for chemoprophylaxis, 137 completed the treatment. A total of 24 tuberculosis cases were identified by September 2003.

### Tuberculin skin tests

The TST was administered by injecting 0.1 mL of phosphate buffered saline containing 0.05 µg tuberculin purified protein derivative (Nippon BCG Manufacturing, Tokyo, Japan) intradermally into the volar surface of the forearm (the Mantoux method). The longest and shortest diameters of erythema and those of induration were read in millimeters 48 hours after the injection. The induration size was determined using the palpation technique. To avoid interobserver variability in administering and reading the test, only one examiner administered and read all the tests done at the public health center.

### Criteria on referral for chemoprophylaxis

We developed the exposure group-specific chemoprophylaxis eligibility criteria based on the erythema measurement with reference to a document issued by the Japanese Ministry of Health and Welfare in 1989.^7^ For convenience, we categorized the criteria into 5 mm intervals. The criteria also considered the increase in erythema size between that which occurred at the regular school-entry checkup and that which occurred during the contact investigation. Details of the criteria are presented in our previous report^11^ and Table 3 of the present report.

### Statistical analysis

The present analysis used the longest diameter of each type of skin reaction. The interrelationship between erythema and induration was examined in the 543 students who had a TST conducted at the public health center. The proportion of students who had tuberculosis disease was computed for all the 566 students for whom the public health center administered the contact investigation. For each exposure group, the computations were performed by stratifying the study subjects according to having had a TST in the contact investigation and being eligible for chemoprophylaxis. Students with false-positive test results, as well as those with false-negative test results (that is students who developed active tuberculosis but were judged as not-infected on TST) in the contact investigation were further analyzed to evaluate the diagnostic accuracy of erythema measurement following TST. The statistical analysis was performed with SPSS^®^ 11.0 (SPSS Inc., Chicago, IL, United States).

## RESULTS

Of the 566 students, 208 (37.8 %) had been revaccinated with BCG at entry into junior high school. Their results of TST responses during infancy had been collected in the regular school-entry checkup and no students had experienced natural occurrence of tuberculin conversion. Therefore, all of the students were considered to have been vaccinated with BCG during infancy.

### Distribution and relationship of TST responses

As shown in Table 1, the mean (standard deviation) response was 33.8 (21.0) mm for erythema and 17.0 (6.1) mm for induration. The mean size of each type of skin response increased with increasing exposure to the index case.

**Table 1.** Descriptive statistics of the 2 skin reactions to the tuberculin skin test obtained during the contact investigation by exposure group.

Exposure group*	Number of subjects^†^	Erythema		Induration	
	
		Mean (mm)	Standard deviation (mm)	Mean (mm)	Standard deviation (mm)
1	28	52.9	16.3	23.3	3.9
2	67	49.5	27.8	20.1	7.0
3	97	39.8	22.8	17.4	7.4
4	351	28.1	15.9	15.8	5.1

All	543	34.1	21.0	17.0	6.1

* : Group 1, homeroom classmates; Group 2, students who shared special classes or extracurricular activities with the case; Group 3, third-grade students after excluding those who belonged to exposure group 1 and group 2; Group 4, first- or second-grade students after excluding those who belonged to exposure group 2.

† : Students who had the tuberculin skin test administered by the public health center are used.

**Figure1.**
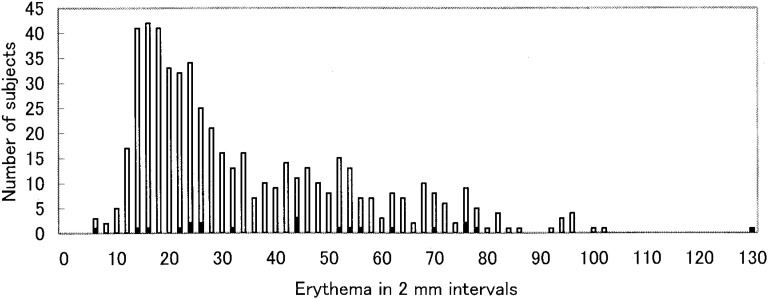
Distribution of erythema by occurrence of tuberculosis (N=543).

**Figure2.**
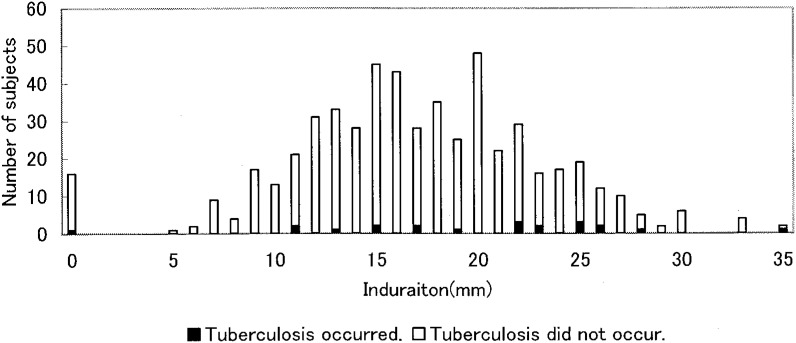
Distribution of induration by occurrence of tuberculosis (N=543).

Distribution of the erythema and induration seen in all subjects are shown in Figures 1 and 2. The erythema diameter ranged from 0 to 129 mm with two peaks: a higher peak at approximately 16 mm and a lower peak at approximately 52 mm. The induration diameter ranged from 0 to 35 mm and it also had two peaks at around 15 mm and 20 mm.

**Figure3.**
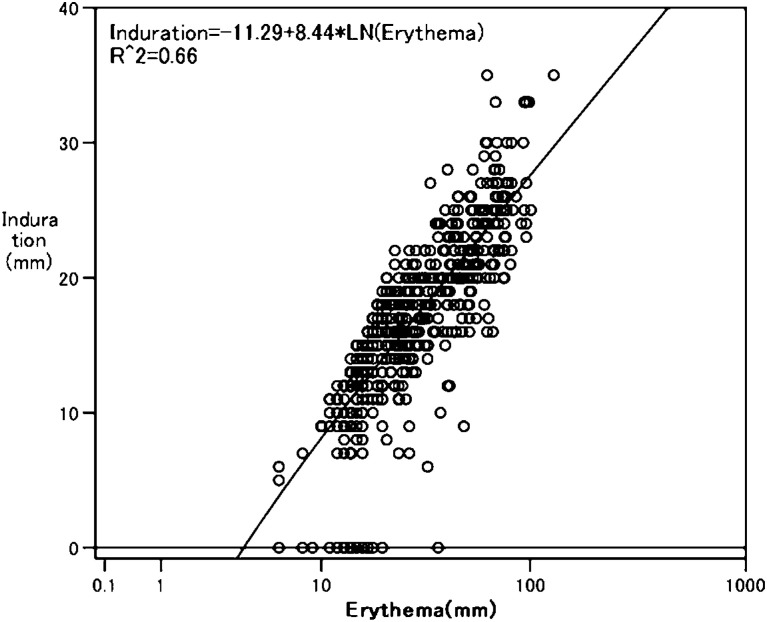
Scatter plot of erythema measurement and induration measurement (N=543).

Figure 3 shows that the logarithm of erythema size was linearly related to the induration size [Induration size = −11.29 + 8.44×ln(erythema size)]. The correlation coefficient between the two types of skin responses was 0.81. This regression analysis shows that erythema size increases exponentially with increasing induration size.

### Incidence proportion of tuberculosis disease and infection

Table 2 shows the incidence of tuberculosis by exposure group based on whether the student had a positive TST in the contact investigation and was eligible for chemoprophylaxis. Eleven cases were identified before or during the contact investigation; five cases occurred in 144 students who were referred for chemoprophylaxis; and eight cases occurred in 411 students that were not required to receive chemoprophylaxis.

**Table 2.** Tuberculosis incidence by subgroup of subjects defined by detection method and eligibility for chemoprophylaxis by exposureg roup.*

Subgroup of subjects (defined by detection method and eligibility for chemoprophylaxis)		Number of subjects observed	Subjects who developed tuberculosis		Number of bacteriologically positive cases

			Number	% of the subjects observed	
Group 1					
Subjects with tuberculosis diagnosed before having the contact investigation		1	1	100	0
Subjects who had the contact investigation	Subjects with tuberculosis diagnosed in the contact investigation	6	6	100	2
	Subjects who were eligible for and completed chemoprophylaxis	19	2	10.5	1
	Subjects who were eligible for and did not complete chemoprophylaxis	1	0	0	0
	Subjects who were not required to receive chemoprophylaxis	3	0	0	0
Total		30	9	30.0	3

Group 2					
Subjects with tuberculosis diagnosed before having the contact investigation		1	1	100	0
Subjects who had the contact investigation	Subjects with tuberculosis diagnosed in the contact investigation	2	2	100	0
	Subjects who were eligible for and completed chemoprophylaxis	40	0	0	0
	Subjects who were eligible for and did not complete chemoprophylaxis	1	0	0	0
	Subjects who were not required to receive chemoprophylaxis	28	3	10.7	1
Total		72	6	8.3	1

Group 3					
Subjects with tuberculosis diagnosed before having the contact investigation		0	-	-	-
Subjects who had the contact investigation	Subjects with tuberculosis diagnosed in the contact investigation	0	-	-	-
	Subjects who were eligible for and completed chemoprophylaxis	43	1	2.3	0
	Subjects who were eligible for and did not complete chemoprophylaxis	4	1	25.0	1
	Subjects who were not required to receive chemoprophylaxis	57	3	5.3	2
Total		104	5	4.8	3

Group 4					
Subjects with tuberculosis diagnosed before having the contact investigation		0	-	-	-
Subjects who had the contact investigation	Subjects with tuberculosis diagnosed in the contact investigation	1	1	100	0
	Subjects who were eligible for and completed chemoprophylaxis	35	0	0	0
	Subjects who were eligible for and did not complete chemoprophylaxis	1	1	100	1
	Subjects who were not required to receive chemoprophylaxis	323	2	0.6	1
Total		360	4	1.1	2

All groups					
Subjects with tuberculosis diagnosed before having the contact investigation		2	2	100	0
Subjects who had the contact investigation	Subjects with tuberculosis diagnosed in the contact investigation	9	9	100	2
	Subjects who were eligible for and completed chemoprophylaxis	137	3	2.2	1
	Subjects who were eligible for and did not complete chemoprophylaxis	7	2	28.6	2
	Subjects who were not required to receive chemoprophylaxis	411	8	1.9	4
Total		566	24	4.2	9

*: Group 1, homeroom classmates; Group 2, students who shared special classes or extracurricular activities with the case; Group 3, third-grade students after excluding those who belonged to exposure group 1 and group 2; Group 4, first- or second-grade students after excluding those who belonged to exposure group 2.

When the students who were referred for chemoprophylaxis were included in the group who had tuberculosis infection,^7^ the number of students infected with *M. tuberculosis* was 163 (144 students who were eligible for chemoprophylaxis; 11 cases who were identified before or during the contact investigation; and eight cases that occurred in students who were not required to receive chemoprophylaxis). The proportion of infected cases was 28.8% (163/566). The proportions by exposure groups were: 90% (27/30) in group 1; 65% (47/72) in group 2; 48% (50/104) in group 3; and 11% (39/360) in group 4. Thus, there was an increase in the proportion of the infected contacts with increasing exposure to the index case.

Of the 144 students who were eligible for chemoprophylaxis, 137 students received the treatment. The risk of developing the disease among students who completed the treatment was less than one-thirteenth of that observed among students who did not receive chemoprophylaxis (2.2 % vs 28.6 %).

### Diagnostic accuracy of erythema measurement

As shown in Table 3, candidates for chemoprophylaxis were chosen on the basis of the size of the erythema response following TST during the contact investigation and by examining the difference in erythema that occurred following TST administration at the regular school-entry checkup and following TST administration during the contact investigation. No students who developed a large erythema response during the contact investigation but in whom the difference from the regular school-entry checkup was small developed tuberculosis. Five such students in groups 2 and 3 developed 30+ mm of erythema during the contact investigation, but had only an increase of 19 mm or smaller in erythema from the previous TST, while six such students in group 4 developed 50-59 mm of erythema during the contact investigation but only had an increase of 29 mm or smaller in erythema from the previous TST.

**Table 3.** Erythema-based referral criteria for chemoprophylaxis by exposure group and tuberculosis incidence in each stratum of the criteria.*

Exposure group^†^	Erythema in the contact investigation (mm)	Increase in erythema (mm)^‡^	Chemoprophylaxis referral criteria	Number of subjects observed^§^	Tuberculosis incidence	

					Number	%
1	30+	Any	Referred	20	2	10.0
	<30	Any	Not referred	2	0	0

2	30+	20+	Referred	40	0	0
	30+	<20	Not referred	2^¶^	0	0
	<30	Any	Not referred	27	3	11.1

3	30+	20+	Referred	47	2	4.3
	30+	<20	Not referred	3	0	0
	<30	Any	Not referred	49	3	6.1

4	60+	Any	Referred	21	1	4.8
	50-59	30+	Referred	14	0	0
	50-59	<30	Not referred	6	0	0
	<50	Any	Not referred	315	2	0.6

* : Subjects with tuberculosis diagnosed before or in the tuberculin skin test were not used in the analysis.

† : Group 1, homeroom classmates; Group 2, students who shared special classes or extracurricular activities with the case; Group 3, third-grade students after excluding those who belonged to exposure group 1 and group 2; Group 4, first- or second-grade students after excluding those who belonged to exposure group 2.

‡ : Increase in the erythema size from the regular school-entry checkup to the contact investigation.

§ : One subject in the exposure group 1, 5 subjects in the exposure group 3, and 3 subjects in the exposure group 4, all of whom did not receive chemoprophylaxis, were excluded because of missing values for erythema measurement at school entry.

¶ : One subject received chemoprophylaxis prescribed by a physician at a medical facility.

Table 4 describes the TST results obtained during the contact investigation of eight students who were not referred for chemoprophylaxis but who did develop tuberculosis during the follow-up period (false-negative test results). In these students, the erythema ranged in size from 6-26 mm, while the induration ranged from 0-19 mm. According to the relationship between the two reactions (described above), 30 mm of erythema (the cut-off point for exposure groups 1-3) corresponded to 17.4 mm of induration, while 50 mm of erythema (the cut-off point for exposure group 4) corresponded to 21.7 mm of induration. The induration sizes of these eight cases with false-negative results were below the values of induration required for referral for chemoprophylaxis.

**Table 4.** Tuberculin skin test (TST) results of 8 subjects who were not required to receive chemoprophylaxis and developed tuberculosis during the follow-up period (cases with false-negative test results).

Number of false-negative case	Exposure group*	Sex	Age (year)	Erythema on the TST at entry into school (Time 1) (mm)	Reactions on the TST in the contact investigation (Time 2) (mm)		Increase in the erythema from Time1 to Time2 (mm)

					Erythema	Induration	
1	2	Female	15	3	24	17	21
2	2	Male	15	12	25	15	13
3	2	Female	13	16	16	11	0
4	3	Male	15	6	23	15	17
5	3	Male	15	13	26	11	13
6	3	Female	15	23	6	0	-17
7	4	Female	13	6	22	19	16
8	4	Female	13	15	14	13	-1

* : Group 2, students who shared special classes or extracurricular activities with the case; Group 3, third-grade students after excluding those who belonged to exposure group 1 and group 2; Group 4, first- or second-grade students after excluding those who belonged to exposure group 2.

## DISCUSSION

A two-modal frequency distribution was detected for both the size of the erythema and the size of the induration responses following TST. This is the characteristic distribution of the skin response following TST when a population with BCG vaccination is exposed to an infectious tuberculosis case.^6^^,^^13^ The natural logarithm of the erythema measurement was linearly correlated with the induration measurement. This finding indicates that erythema is equal to induration as a way to identify a positive tuberculin reaction resulting from *M. tuberculosis*.

We developed chemoprophylaxis referral criteria based on the erythema measurement obtained during the contact investigation and the changes in the erythema seen between two time points (the regular school-entry checkup and the contact investigation). There is no gold standard that is used to diagnose tuberculosis infection, so that the exact values of sensitivity, specificity, and predictive values of a positive and a negative test result could not be computed in the present study. Therefore, the rationale for choosing the particular cut-off points of erythema measurement used in this study need to be clarified. In practice, when detecting a tuberculosis infection it is important to be specific enough to reduce the number of false-positive cases who are referred for unnecessary chemoprophylaxis. In the present study, we chose the cut-off points for the size of the erythema with special attention to this requirement. Among BCG vaccinated persons with smear-positive tuberculosis, erythema measurement is reported to show a unimodal distribution with a mode at 30 mm.^9^ This is the cut-off point that was used for students who had close contact with the index case (students of groups 1-3). However, the cut-off point was raised to 50 mm for the group of contacts who could be expected to have a low prevalence of tuberculosis infection, which is students of group 4 whose contacts with the index case were less intense. Thus, we raised the cut-off point in group 4 to keep the predictive value of a positive test result at a level similar to that of the other groups that had higher prevalence levels of tuberculosis infection.

By incorporating the difference between the erythema responses that occurred at two time points, the specificity of the test was improved. In BCG-vaccinated persons who have a TST after exposure to *M. tuberculosis*, and who show an increase of 15+ mm of induration over the induration observed on a TST done before the exposure, it is probable that this is associated with newly acquired *M. tuberculosis*.^14^ According to the relationship between the two skin reactions seen in the present study, an increase of 15+ mm of induration corresponded to an increase of 22.5+ mm of erythema. Thus, a 20 mm difference in erythema was used as the cut-off point for groups 2 and 3. When this cut-off point was added to the criterion, five students in the two groups who otherwise would have been referred for chemoprophylaxis were judged as not-infected. Thus, the present study indicates that a cut-off point based on the difference in erythema between pre- and post-exposure TST can improve the specificity when using erythema to determine who should receive chemoprophylaxis.

However, as inevitably happens, when cut-off points are set, false-negative test results are unavoidable. According to the relationship between the exposure group specific cut-off points chosen in the present study and the mode of erythema distribution in persons with tuberculosis disease described above, the cut-off point seemed to be able to detect only about half of the students with tuberculosis infection in groups 1-3 and less than half of such students in group 4. Thus, measures that deal with subjects who have false-negative test results must be incorporated into any control program. Recently, an *in vitro* whole blood interferon-*γ* assay to measure cell-mediated responses to *M. tuberculosis*-specific antigens has been demonstrated to be highly specific and sensitive for detecting infection.^15^^,^^16^ However, until such blood tests become available for use in practice, the TST remains the only available test to detect latent tuberculosis infection. Thus, when tuberculosis infection occurs in a population with BCG vaccination, in anticipation of the occurrence of false-negative test results for tuberculosis infection one must administer periodic chest X-rays and symptom screening for at least 2 years, during which the cumulative incidence of active tuberculosis reaches 80%.^13^^,^^17^

There are some limitations of the present study. Firstly, the measurement of skin reactions might have been influenced by the bias and the variability of the single examiner who determined all of the results. Variability appears to be a major contributing cause to the imperfect correlation between erythema and induration. However, it is unlikely that the direction and degree of bias were different between the two measurements for this examiner. Therefore, the bias would not appear to have systematically distorted the observations. Secondly, the criteria for tuberculosis infection developed in the present study have limited applicability. The recommended procedure for a contact investigation is to do two TSTs at a two-month interval (immediately after the notification of the index case and two months after the notification).^7^ However, the present study conducted only a single TST two months after the notification of the index case^18^ and used the TST results at school-entry as a surrogate measure to reflect the cellular immunity level before exposure. Skipping the first test might help minimize false positive cases resulting from boosting occurring as a result of the first test.^5^ However, surrogate information is no longer available because a revised law abolished the BCG revaccination program at school-entry.

The present findings indicate that measuring erythema is a valid way to interpret TST results. Given the difficulties and variability in obtaining induration measurements, it is useful to conduct a tuberculosis prevention and control program based on measuring the amount of erythema following a TST, in contacts in whom the erythema can be easily observed, such as the Japanese population.
